# Prehospital ventilation strategies in out-of-hospital cardiac arrest: A protocol for a randomized controlled trial (PIVOT trial)

**DOI:** 10.1016/j.resplu.2024.100827

**Published:** 2024-11-16

**Authors:** Cheng-Yi Fan, Sih-Shiang Huang, Chi-Hsin Chen, Chih-Wei Sung, Chin-Hao Chang, Tung-Hsiu Hung, Yen-Chen Liu, Edward Pei-Chuan Huang

**Affiliations:** a Department of Emergency Medicine, National Taiwan University Hospital Hsin-Chu Branch, Hsinchu, Taiwan; b Graduate Institute of Biomedical Informatics, College of Medical Science and Technology, Taipei Medical University, Taipei, Taiwan; c Department of Emergency Medicine, College of Medicine, National Taiwan University, Taipei, Taiwan; d Department of Medical Research, National Taiwan University Hospital, Taiwan; e Fire Bureau of Hsinchu County, Hsinchu, Taiwan; f Taiwan Police College, Taiwan; g National Yang Ming Chiao Tung University, Taiwan; h Department of Emergency Medicine, National Taiwan University Hospital, Taipei, Taiwan

**Keywords:** Out-of-hospital cardiac arrest, Mechanical ventilation, Bag-valve-mask, Emergency medical service

## Abstract

**Aims:**

The PIVOT trial evaluates the clinical outcomes and ventilatory quality of an automatic pneumatic ventilation method compared to a bag-valve-mask ventilation method in patients who have experienced out-of-hospital cardiac arrest and have had an advanced airway placed.

**Methods:**

The PIVOT trial is a pragmatic, open-label, multicenter randomized controlled trial. It aims to recruit 514 patients in Hsinchu County, Taiwan. Adult, non-trauma patients who experience out-of-hospital cardiac arrest, are treated by emergency medical services, and have an advanced airway in place will be randomized. Biweekly cluster randomization will assign EMS teams to either the automatic pneumatic ventilation group or the bag-valve-mask group. Informed consent is waived. The primary outcome is the return of spontaneous circulation, either prehospital or in-hospital. Secondary outcomes include survival to discharge, neurological outcomes, prehospital ventilatory quality, and the content of prehospital resuscitation. Participants will be followed until they pass away or are discharged from the hospital.

**Conclusion:**

The PIVOT trial will provide new insight on the clinical effectiveness of automatic pneumatic ventilation in patients experienced out-of-hospital cardiac arrest.

**Trial number**: NCT06067204 in *clinicaltrial.gov*

## Introduction

Out-of-hospital cardiac arrest (OHCA) is a critical issue in the fields of public health and emergency medicine, with an annual incidence rate of 51.1 cases per 100,000 population in Taiwan^1^. The survival and prognosis of OHCA patients are influenced by numerous factors. The American Heart Association's chain of survival encompasses pre-hospital care, advanced cardiac life support (ACLS), and post-survival recovery and rehabilitation.^2^ With the professionalization of emergency medical technicians and the promotion of bystander interventions, the importance of pre-hospital emergency care has become increasingly evident. Current literature confirms that high-quality chest compressions and early defibrillation improve OHCA patient outcomes.^3^, ^4^ However, discussions on ventilation methods, devices, or strategies remain extensive and inconclusive.

In Basic Life Support (BLS), a 30:2 ratio of chest compressions to ventilations is emphasized. In ACLS, after establishing an advanced airway, the protocol shifts to providing one breath every six seconds, independent of chest compressions. Recent research on ventilation has primarily focused on establishing advanced airways. Practically, options include the bag-valve-mask (BVM), supraglottic airway (SGA), and endotracheal tube (ETT), with no clear consensus on their relative impact on patient outcomes.^5^ Regardless of the airway management method, stable oxygen delivery requires manual effort, which can burden emergency medical technicians performing other ACLS tasks. Insufficient ventilation can lead to hypoxemia, hypercapnia, acidosis, or atelectasis,^6^ while over-ventilation can result in air trapping, increased intrapulmonary pressure, reduced venous return, decreased cardiac output, and reduced coronary perfusion.^5^, ^7^ Thus, providing a stable and convenient ventilation method is a current priority.

Automatic ventilation devices are classified into electronic and pneumatic types. The former relies on a power supply, while the latter operates purely mechanically. Portable ventilators can achieve similar outcomes to manual BVM ventilation,^8^ enabling emergency medical technicians to complete more tasks and documentation.^9^ However, ventilators are more complex to operate, requiring specific training and multiple parameter settings. Crucially, there is no consensus or guideline on ventilator settings (such as ventilation mode, tidal volume, rate, and inspiratory-to-expiratory ratio) for OHCA patients in pre-hospital settings.^10^ Automatic pneumatic ventilators (APVs) are simpler to use, with manual and automatic modes, and are small, portable, and cost-effective. Historically, the American Heart Association recommended against their use in OHCA patients due to the risk of increased positive end-expiratory pressure (PEEP).^11^ Nonetheless, recent small-scale studies have shown that APVs can achieve similar oxygenation and ventilation effects as BVMs,^12^, ^13^ without significantly increasing PEEP or causing high peak inspiratory pressure,^14^ and can reduce the risk of patient gastric regurgitation.^12^, ^15^ However, current literature does not confirm that APV use improves patient outcomes, and there is a lack of large-scale randomized controlled trials to verify the impact of APVs on various aspects of pre-hospital care for OHCA patients. This study aims to conduct a cluster randomization trial in Hsinchu County to compare the effects of APVs and BVMs on the prognosis of OHCA patients after advanced airway establishment.

## Methods

PIVOT trial is a pragmatic, open-label, multi-center randomized controlled trial. The trial was registered in Clinicaltrial.gov as NCT06067204. The first patient was recruited on October 2nd, 2023. The planned ending date of trial is on December 31st, 2025.

The trial is based on the emergency medical service (EMS) in Hsin-chu County, Taiwan and currently recruiting patients. Initially, eight EMS teams participated, and then three others joined later. These patients were sent to the nearby and appropriate hospitals in the region.

**Fig. 1:**
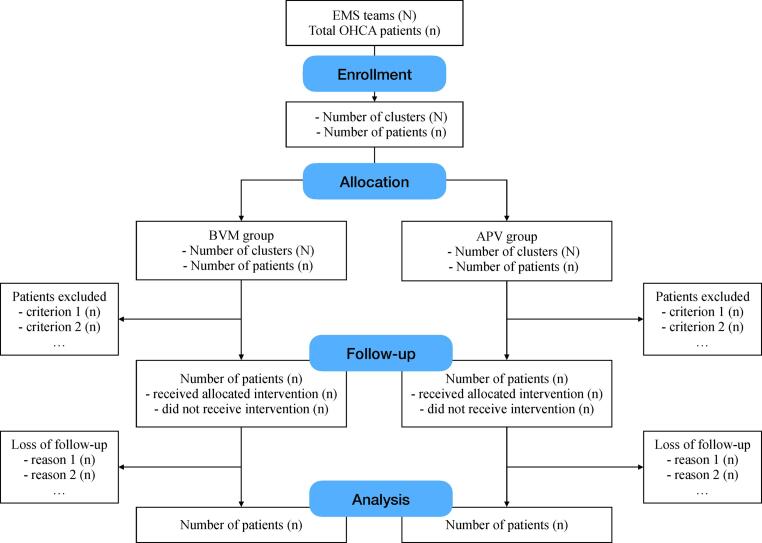
PIVOT trial CONOSRT diagram. APV: automated pneumatic ventilator; BVM: bag-valve-mask; OHCA: out-of-hospital cardiac arrest; ROSC: return of spontaneous circulation.

The trial was designed in accordance with the declaration of Helsinki. The protocol was developed and modified from the Emergency Medical Technician Operating Procedure Manual from the local EMS. National Taiwan University sponsored (grant no. 113-S0057) and the Institutional Review Board approved the trial (no. 202304132RINB). The trial will be reported in accordance with the CONSORT (Consolidated Standards of Reporting Trials) reporting recommendation. The flow chart of CONSORT is demonstrated in Fig. 1.

### Trial objectives

This trial’s primary objective is to evaluate the clinical effectiveness of APV method in prehospital OHCA resuscitation compared to BVM method. The primary outcome is any return of spontaneous circulation (ROSC).

The secondary objectives include:

1. To compare the parameters of ventilation between APV and BVM methods
2. To evaluate the overall resuscitation quality in APV and BVM groups
3. To compare the satisfaction of emergency medical technician between APV and BVM groups.

### Eligibility criteria

The trial included adult patients have cardiac arrest before EMS arrival, during on scene resuscitation or in transportation to the hospital. Patients are excluded if they meet the following criteria:

1. Pregnant,
2. Died from trauma,
3. ROSC before EMS arrival,
4. Dead on arrival (reaching conditions such as decomposition, rigor mortis, severe burns, decapitation, evisceration, or trunk fracture), or
5. Refusal of medical treatment by family members
6. No placement of an advanced airway throughout the procedure

### OHCA resuscitation procedures and patient enrollment

In Taiwan, the EMS system is activated by an emergency phone call, which is processed by a dispatcher. The dispatcher assesses the situation at the scene and attempts to identify OHCA with the help of the caller. If OHCA is confirmed or suspected, the dispatcher provides instructions for dispatcher-assisted CPR and alerts the EMTs. Upon arrival at the scene, the EMTs re-evaluate the patient’s vital signs. If cardiac arrest is confirmed, the patient will be screened for study eligibility.

The CPR is initiated immediately unless the family refuse for any medical treatment or the patient was dead on arrival. An AED is applied to determine if the cardiac rhythm is shockable or non-shockable. After recording the initial rhythm and administering defibrillation if needed, *an advanced airway will be placed (endotracheal tube or supraglottic airway)*, provided there are no contraindications. Contraindications include family’s refusal of invasive airway management or trismus. After an advanced airway is established, it is connected to either an APV or a BVM, based on the randomization protocol. Once the ventilation setup is done, EMS personnel proceed with the insertion of an intravenous catheter or intraosseous catheter and administer epinephrine as part of the resuscitation process. EMS interventions cease upon hospital admission, at which point the receiving hospital continues advanced cardiovascular life support resuscitation efforts.

### Study intervention

Eligible participants are randomized to the APV method or BVM method group.

For patients randomized to the APV method group, once the advanced airway is secured, the APV will be activated to provide ventilations. The rate and volume of every ventilation are controlled by the APV, which are set at 10 times per minute and 500–600 ml. This prespecified tidal volume is based on the current American Heart Association guidelines on CPR.^16^ The EMTs can decide the volume between this range according to the patients’ body size. No positive end-expiratory pressure was applied. The pressure limit is 60 cmH_2_O.

For patients randomized to the BVM group, once the advanced airway is secured, the EMS will deliver ventilations manually with the BVM. The rate and volume of every ventilation are controlled by the technicians, which is 10 times per minute and 1/2 to 2/3 of the Ambu bag volume. No positive end-expiratory pressure was applied. The pressure limit is 60 cmH_2_O. The ventilation parameters (ventilation rate, tidal volume and end-tidal carbon dioxide) are measured by Zoll X-series, which is the standard equipment in all participating EMS teams.

The technicians will change to the other method if either of the equipment failed to ventilate the patients. The randomized intervention will stop upon arrival at the hospital and be handed over to the in-hospital resuscitation team. However, if the patient achieves ROSC during transportation, the trial intervention will continue until hospital arrival.

### Trial outcomes

Trial outcomes included two aspects, the first is related to patient outcome: ROSC, short-term survival and neurological function. The second is related to prehospital resuscitation by the EMS including the procedures and satisfaction in the dispatch. The list of outcomes was developed in collaboration with the EMS technicians from different levels.

The primary outcome of the trial is any ROSC, including prehospital ROSC or ROSC achieved after in-hospital resuscitation.

The secondary patient outcomes are:

• Sustained ROSC in 24 h
• Survival to hospital discharge
• Favorable neurological outcome after discharge

The secondary outcomes for prehospital resuscitation are:

• Chest compression fraction
• Chest compression depth and rate
• Intravenous catheter placement
• Epinephrine injection
• Ventilation parameters (rate, tidal volume and end-tidal carbon dioxide)
• Satisfaction of EMS technician during the resuscitation

The safety profile of the patients will also be reported:

• Pneumothorax associated with study’s intervention

### Participant recruitment and randomization

Every EMS technician participated in the trial will be trained with the study protocol and the standard procedure with APV and BVM for 2 h. The EMS teams will have a 2-month test run phase before officially enroll in the trial. The decision to recruit patients to the trial is made by the EMS technicians.

The participating EMS teams were divided into two randomized clusters by a research assistant. The randomization sequence was generated using a tool provided by Sealed Envelope Ltd. (2022).^17^ Each cluster was assigned to either the APV or BVM group, with each cluster lasting 14 days (two weeks) and a block size of six clusters. Before the trial officially begins, we simulated the randomization sequence generation process for multiple time to ensure a 1:1 ratio between the two clusters. Only the trial assistant keeps the whole randomization sequence.

Every new group allocation begins on every other Monday 8 AM. The allocations were assigned to each EMS team 3 days before via social network application.

### Blinding

Blinding of the ventilator method to the EMS team is not feasible due to the study nature. The hospital staffs who take over the patient in the emergency room will be aware of the ventilation device being used. Participants will be unaware of the treatment because they are unconscious during OHCA resuscitation.

### Consent, data collection and follow-up

The PIVOT trial recruits unconscious patients who have experienced OHCA, and the prehospital treatment is time-sensitive. Therefore, it is challenging and impractical for EMS technicians to obtain informed consent from either the patient or their family members before initiating ACLS and trial interventions. Following discussions with the Institutional Review Board (IRB), the trial has been approved for a waiver of informed consent for all participants.

Most variables will be collected from the rescue reporting form, which is documented on-site by the EMS technicians. In addition, the EMS technicians will complete a satisfaction questionnaire using a 5-point Likert scale (highly disagree, disagree, neutral, agree, highly agree) every time they successfully recruit a patient. Each EMS technician will receive a gift card as compensation for completing the questionnaire. Prognostic outcomes will be gathered from the reports of the hospitals where the patients are treated. Follow-up on these outcomes will occur monthly until the patient either passes away or is discharged. Ventilation and chest compression feedback data will be exported from the Zoll X-series in.csv format, and these files will be uploaded to the REDCap database on a weekly basis.

### Sample size calculation

During the study, we recalculated the sample size based on the pilot study by Shin et al., which compared automatic mechanical ventilation (MV) with manual bag ventilation (BV) during CPR.^18^ In that study, the primary outcome, any ROSC, was achieved in 56.7 % of the MV group and 43.3 % of the BV group—values closely aligning with our expected ROSC rates. Thus, we conducted an initial power analysis in G*Power 3.1 using the following parameters: Z test family, difference between two independent proportions, two-tailed test, proportions of 0.567 and 0.433, alpha of 0.05, power of 0.8, and equal allocation (1:1). This analysis suggested 218 patients per arm, totaling 436.

To account for the clustering effect due to randomization at the EMS team level, we included an intraclass correlation coefficient (ICC). Our EMS teams are expected to recruit 3 patients per cluster (over 14 days). Given the substantial variability in OHCA patient conditions, we assumed a low ICC of 0.06. The design effect was therefore calculated as 1+(3 − 1) × 0.06 = 1.12, adjusting our sample size to 488. Finally, with an estimated 5 % dropout rate, the final required sample size was increased to 514 patients.

### Statistical analysis

Statistical analyses in this study will be performed using SPSS (version 26.0, IBM Corp., Armonk, NY, USA) with an intention-to-treat approach. Descriptive statistics will present demographic data, ventilation parameters, and outcomes for the APV and BVM groups, with categorical variables shown as counts (percentages) and continuous variables as medians (interquartile range). Categorical variables will be compared using Pearson’s chi-square or Fisher’s exact test, while the Mann–Whitney *U* test will be used for continuous variables. Multivariable logistic regression and generalized estimating equations (GEE) will assess the association between ventilation methods and patient outcomes, adjusting for confounders and accounting for clustering within EMS teams. A subgroup analysis, illustrated in a forest plot, will explore the intervention impact across subgroups. Additionally, the linear mixed-effects model (LMM) will compare ventilation parameters between groups, with demographics and resuscitation management as fixed effects and patients as random effects. Statistical significance will be set at p < 0.05, with Bonferroni correction applied in post-hoc analyses.

### Safety monitoring

This study has established a Data and Safety Monitoring Board (DSMB) following NIH standards, comprising experts in emergency medicine, EMS and a statistician. The DSMB will review data completeness and monitor patient safety every six months. An independent interim analysis will be conducted when enrollment reaches 50 % of the targeted number of participants. If concerns arise, the DSMB will consult with the principal investigator to determine whether the study should be suspended. The stopping criteria are listed in the supplementary file.

### Data sharing

The trial investigators will open for data sharing request six months after the primary results are published. Requests should be directed to the corresponding author of the primary results paper and must include a detailed plan for data use. The requests will be reviewed by the trial administrating team.

### Dissemination

The results of the trial are intended to be disseminated among EMS personnel, emergency clinicians, policymakers, and the public. Dissemination strategies will include publication in peer-reviewed journals, presentations at domestic and international clinical conferences, press releases, and social media. We hope the trial’s findings will shed light on the prehospital ventilatory strategies, an area that has received less attention in resuscitation guidelines.

## Discussion

### Hypothesis for Improving ROSC

The primary hypothesis of this study is that the use of APV OHCA may improve the ROSC by ensuring consistent, controlled ventilation. APV can prevent common errors associated with manual BVM ventilation, such as hyperventilation or hypoventilation, both of which are detrimental to patient outcomes. Hyperventilation, for instance, can lead to increased intrathoracic pressure, decreased venous return, and reduced coronary perfusion, negatively impacting the chances of ROSC. By maintaining optimal ventilation parameters, APV creates a stable physiological environment conducive to successful resuscitation.

Furthermore, the benefits of APV are hypothesized to extend beyond ventilation alone. By automating this process, APV frees up an EMT, allowing them to focus on other critical resuscitation tasks, such as establishing intravenous (IV) or intraosseous (IO) access, administering life-saving medications, or using pre-hospital ultrasound to assess the underlying cause of the arrest. This reallocation of resources and manpower may enhance the overall quality of the resuscitation process and contribute to better OHCA prognosis.

### Generalizability and anticipated challenges

While the Taiwan EMS system is unique in its centralized structure, its core practices for OHCA management—such as CPR, defibrillation, and airway management—are globally applicable. The equipment used, including the APV device, is internationally available, making the study findings relevant to other professional EMS systems, particularly in high-income countries. However, differences in EMS infrastructure, particularly in settings where volunteer-based systems predominate, may affect generalizability.

One of the primary challenges anticipated is ensuring that EMS personnel are proficient with the APV device. Although the device is designed for ease of use, any new technology requires training. To address this, we implemented a comprehensive two-hour training session to ensure that every EMT can set up the ventilation device within one minute after placing an advanced airway. Following the training, a two-month trial run will be conducted to familiarize EMS teams with the trial process and device before the full trial initiation. Additionally, potential device failure during resuscitation poses a risk. To mitigate this, we have included a contingency protocol allowing EMTs to switch to manual BVM ventilation if necessary, ensuring uninterrupted patient care.

Finally, protocol adherence is critical, especially in high-stress resuscitation scenarios. EMTs may be inclined to revert to familiar manual techniques. To counter this, we incorporated continuous monitoring and refresher sessions to reinforce the use of APV. We also assess the impact on EMT workload through technician satisfaction surveys, ensuring that APV improves, rather than complicates, task management during resuscitation.

## Conclusion

The choice of using automatic or manual ventilation after advanced airway is established on OHCA patient is still uncertain. The PIVOT trial is a pragmatic, open-label, multicenter randomized controlled trial that will investigate the prognostic outcomes and ventilatory quality in these two strategies in adult OHCA.

## Author statement

All authors contributed substantially to this work. Chih-Wei Sung and Edward Pei-Chuan Huang originally conceived of the project. Tung-Shiu Hung and Yen-Cheng Liu coordinate the resources in the fire bureau. Cheng-Yi Fan, Chi-Hsin Chen and Sih-Shiang Huang design the trial. Cheng-Yi Fan wrote the manuscript. Chin-Hao Chang design the statistical analysis. Edward Pei-Chuan Huang and Chih-Wei Sung gave critical revisions. All authors have read and approved this manuscript.

## CRediT authorship contribution statement

**Cheng-Yi Fan:** Writing – original draft, Investigation, Data curation, Conceptualization. **Sih-Shiang Huang:** Investigation, Data curation. **Chi-Hsin Chen:** Methodology, Investigation, Data curation. **Chih-Wei Sung:** Writing – review & editing, Conceptualization. **Chin-Hao Chang:** Formal analysis. **Tung-Hsiu Hung:** Resources. **Yen-Chen Liu:** Resources. **Edward Pei-Chuan Huang:** Writing – review & editing, Funding acquisition, Conceptualization.

## Funding

This work was funded by National Taiwan University Hospital (grant number: 1113-S0057).

## Declaration of competing interest

The authors declare that they have no known competing financial interests or personal relationships that could have appeared to influence the work reported in this paper.

## Data Availability

Data will be made available on request.

## Appendix A Supplementary material

Supplementary Data 1 Trial Protocol
