# Supplementary material for: Prehospital ventilation strategies in out-of-hospital cardiac arrest: A protocol for a randomized controlled trial (PIVOT trial)
Source: Resusc Plus. 2024 Nov 16;20:100827. doi: 10.1016/j.resplu.2024.100827 (PMC11609668; doi:10.1016/j.resplu.2024.100827)
Supplement: Supplementary Data 1 — Trial Protocol [file mmc1.docx]

Prehospital Ventilation Strategy Trial in Out-of-Hospital Cardiac Arrest (PIVOT Trial)

Version 1.1 November 2024

[I. Acronyms and Abbreviations 2](#_Toc181731054)

[II. Study Board 3](#_Toc181731055)

[III. Trial Summary 5](#_Toc181731056)

[I. Backgrounds 6](#_Toc181731057)

[II. Literature Review 8](#_Toc181731058)

[III. Trial Design 11](#_Toc181731059)

[A. Hypothesis 11](#_Toc181731060)

[B. Objective 11](#_Toc181731061)

[C. Inclusion criteria 11](#_Toc181731062)

[D. Exclusion criteria 11](#_Toc181731063)

[E. Trial setting 12](#_Toc181731064)

[F. Randomization and blinding 13](#_Toc181731065)

[G. Pre-trial training program 13](#_Toc181731066)

[H. Exposure management 13](#_Toc181731067)

[I. Outcome measurement 14](#_Toc181731068)

[J. Study procedures and workflow 14](#_Toc181731069)

[IV. Sample Size Estimation 16](#_Toc181731070)

[V. Data and Safety Monitoring Boards and Interim Analysis 17](#_Toc181731071)

[A. Interim analysis and stopping criteria 17](#_Toc181731072)

[VI. Data Collection and Follow-up 18](#_Toc181731073)

[VII. Data Management 18](#_Toc181731074)

[VIII. Analytic Plan 19](#_Toc181731075)

[IX. Ethics 20](#_Toc181731076)

[X. Dummy Tables 20](#_Toc181731077)

[XI. Timeline 23](#_Toc181731078)

[A. First year working plan (2023) 23](#_Toc181731079)

[B. Second year working plan (2024) 23](#_Toc181731080)

[C. Third year working plan (2025) 23](#_Toc181731081)

[D. Fourth year working plan (2026) 24](#_Toc181731082)

[XII. Reference 24](#_Toc181731083)

# Acronyms and Abbreviations

| ACLS | Advanced cardiopulmonary life support |
| --- | --- |
| APV | Automated pneumatic ventilation |
| ATV | Automatic transport ventilator |
| ABG | Arterial blood gas |
| BVM | Bag-valve-mask |
| CPR | Cardiopulmonary resuscitation |
| COPD | Chronic obstructive pulmonary disease |
| EMT | Emergency medical technician |
| EMT-P | Emergency medical technician - paramedics |
| ETT | Endotracheal tube |
| IO | Intraosseous |
| IV | Intravenous |
| MV | Mechanical ventilation |
| OHCA | Out-of-hospital cardiac arrest |
| RCT | Randomized controlled trial |
| SGA | Supraglottic airway |

# Study Board

1. Trial Administration

| Role | Name | Affiliation |
| --- | --- | --- |
| Trial director | Edward Pei-Chuan Huang | National Taiwan University hospital Hsin-chu Branch |
| Co-director | Yen-Cheng Liu | Fire Bureau of Hsinchu County |
| Co-director | Tung-Shiu Hung | Fire Bureau of Hsinchu County |
| Contact person | Cheng-Yi Fan | National Taiwan University hospital Hsin-chu Branch |

1. Participating Fire Departments

| EMS team | EMT-P | EMT-2 | Joined date |
| --- | --- | --- | --- |
| Hukou Division | 2 | 16 | 2022/10/22 |
| Xingong Division | 1 | 17 |  |
| Xinpu Division | 3 | 12 |  |
| Qionglin Division | 14 | 1 |  |
| Guanmin Division | 10 | 4 |  |
| Zhudong Division | 4 | 19 |  |
| Guanxi Division | 2 | 12 |  |
| Erchong Division | 2 | 11 |  |
| Xinfeng Division | 2 | 10 | 2024/01/01 |
| Fengtien Division | 1 | 13 | 2024/05/01 |
| Hengshan Division | 1 | 11 | 2024/07/01 |

1. Data and Safety Monitoring Boards

| Affiliation | Name | Role |
| --- | --- | --- |
| National Taiwan University Hospital Hsin-chu Branch | Chun-Hsiang Huang | Expert from healthcare system |
| Fire Bureau of Hsin-chu County | Li-Cheng Li | Expert from fire department |
| National Taiwan University Hospital Hsin-chu Branch | Mu-Yang Hsieh | Biostatistics expert |

# Trial Summary

The prognosis of patients experiencing out-of-hospital cardiac arrest (OHCA) is influenced by multiple factors, with pre-hospital emergency care being particularly critical. In addition to high-quality chest compressions and early defibrillation, effective ventilation is also a key component. With the increasing professionalization of emergency medical personnel, the establishment of advanced airways has become more common. However, ventilation still relies on manual operation, and the ability to provide consistent and effective ventilation without interfering with other resuscitation measures is a significant concern. The use of automatic oxygen delivery devices presents a potential solution.

The PIVOT trial will be conducted in fire department units across Hsinchu County, coordinated by National Taiwan University Hospital Hsinchu Branch. It is scheduled to take place between June 1, 2023, and December 31, 2025. Fire department units will be divided into two groups using bi-weekly cluster randomization: a control group using manual ventilation and an intervention group using automatic oxygen delivery devices. The study will include cases of non-traumatic OHCA reported within the region. The primary objective is to analyze the impact of both groups on patient survival and prognosis, while the secondary objective is to evaluate ventilation parameters and other quality management indicators related to OHCA care.

# Backgrounds

Out-of-hospital cardiac arrest (OHCA) is a critical medical emergency with substantial public health implications. The global incidence of OHCA ranges from 50 to 110 per 100,000 population annually [1]. Despite advances in resuscitation techniques, survival rates remain low, with less than 10% of patients surviving to hospital discharge [2]. The economic burden associated with OHCA is considerable, driven by both direct medical costs and indirect costs such as lost productivity, emphasizing the need for improved resuscitation strategies [3, 4].

In the prehospital setting, emergency medical technicians (EMTs) and paramedics are tasked with numerous responsibilities during resuscitation efforts [5]. These include checking vital signs, establishing intravenous or intraosseous access, administering medications and ensuring adequate chest compression and ventilation while transporting the patients. Effective ventilation is particularly challenging, often performed using a bag-valve-mask (BVM) device. Manual BVM ventilation can be inconsistent and labor-intensive, requiring one or more team members to focus solely on this task, which can detract from other critical resuscitation activities [6].

The American Heart Association (AHA) guidelines currently recommend manual BVM ventilation during cardiopulmonary resuscitation (CPR) [7]. However, this method is fraught with challenges, including the risk of hyperventilation, insufficient ventilation, and interruptions during chest compressions. These issues can compromise the quality of resuscitation and negatively impact patient outcomes.

Mechanical ventilation offers potential advantages over manual BVM ventilation. Studies have demonstrated that mechanical ventilators can provide more consistent and controlled ventilation [8]. For instance, Weiss et al. conducted a study comparing an automatic transport ventilator (ATV) with manual BVM ventilation. The results showed that the ATV allowed EMTs to accomplish additional tasks, improve documentation, and enhance overall patient care [9]. Similarly, the SYMEVECA phase 1 trial indicated that mechanical ventilators provided better ventilatory parameters compared to BVM, although the impact on clinical outcomes needs further investigation [8].

Despite these advantages, most research on mechanical ventilation during CPR has focused on in-hospital settings. The use of automated pneumatic ventilators (APVs) in the prehospital environment remains underexplored. APVs are designed to deliver consistent ventilation with minimal manual intervention, potentially freeing up EMTs to perform other critical tasks during resuscitation. This could be particularly beneficial in prehospital settings where resources and personnel are often limited [9].

There is a significant knowledge gap regarding the effectiveness of APVs as a substitute for BVMs in prehospital OHCA management. Key concerns include whether APVs can deliver sufficient ventilation without causing high plateau pressures that may lead to barotrauma. Addressing this gap is crucial for developing evidence-based guidelines that can enhance prehospital care and improve patient outcomes. This study aims to evaluate the effectiveness of APVs compared to traditional BVMs in the prehospital resuscitation of OHCA patients.

# Literature Review

| No. | Author, year | Title | Study design | Outcomes |
| --- | --- | --- | --- | --- |
| 1 | JA Johannigman, 1995  [10] | Out-of-hospital Ventilation: Bag-Valve Device vs Transport Ventilator | A prospective, nonrandomized sampling comparing ABG between transport ventilator and BVM among intubated out-of-hospital airway management patients. | No differences in ABG data between transport ventilator and BVM groups.  SGA had inadequate ventilation compared to the endotracheal tube. |
| 2 | JJ Osterwalder, 1998  [11] | Effectiveness of mask ventilation in a training mannikin. A comparison between the Oxylator EM100 and the bag-valve device | A RCT comparing ventilatory volume and gastric inflation between APV (peak pressure 50 cm-H_2_O) and BVM on an instrumented manikin. | APV had better tidal volume (1196 vs. 556 mL, p<0.01) with minimal risk of gastric inflation. |
| 3 | SJ Weiss, 2005  [9] | Automatic transport ventilator versus bag valve in the EMS setting: a prospective, randomized trial | A RCT comparing ATV (peak pressure 60 cm-H_2_O) and BVM among OHCA patients using questionnaires completed by EMT. | EMTs were able to accomplish more tasks, document more completely, and provide better patient care with the use of the ATV. |
| 4 | TA Barnes, 2005  [12] | Comparison of an oxygen-powered flow-limited resuscitator to manual ventilation with an adult 1,000-mL self-inflating bag | The model study compared the volume delivered to lungs and stomach between the Oxylator and BVM. | The volumes delivered with the Oxylator and the BVM to the lungs and stomach were not significantly different. |
| 5 | Salas N, 2007  [13] | Comparison of ventilation and cardiac compressions using the Impact Model 730 automatic transport ventilator compared to a conventional bag valve with a facemask in a model of adult cardiopulmonary arrest | The randomized crossover quasi-experimental study compared the volume delivered to lungs and stomach between ATV and BVM on the instrumented manikin | There were no differences in tidal volume between 2 groups, but BVM let more air into the stomach (137 vs. 14mL, p<0.05). |
| 6 | X Hu, 2013  [14] | The effects of an automatic, low pressure and constant flow ventilation device versus manual ventilation during cardiovascular resuscitation in a porcine model of cardiac arrest | The pig model study compared the ventilation parameters between APV and BVM. | Ventilation with APV during CPR provides adequate ventilation compared to BVM. |
| 7 | C Kill, 2014  [15] | Mechanical Ventilation During Cardiopulmonary Resuscitation With Intermittent Positive-Pressure Ventilation, Bilevel Ventilation, or Chest Compression Synchronized Ventilation in a Pig Model | The animal study compared the ABG, blood pressure and ROSC between 3 types of ventilation:  intermittent positive-pressure ventilation (IPPV), bilevel ventilation, and chest compression synchronized ventilation | IPPV and bilevel ventilation showed similar ventilation and oxygenation.  Chest compression synchronized ventilation elicited the highest mean arterial pressure and best oxygenation |
| 8 | LBA Neumamm, 2021  [16] | Empirical evidence for safety of mechanical ventilation during simulated cardiopulmonary resuscitation on a physical model. | The manikin study compared the ventilation and peak pressure during chest compression among 3 types of ventilation:  volume-controlled (VCV), pressure-controlled (PCV), pressure regulated volume-controlled (PRVC). | Ventilator use during chest compression resulted in pressure and volume similar to BVM. |
| 9 | S Orlab, 2021  [17] | Reliability of mechanical ventilation during continuous chest compressions: a crossover study of transport ventilators in a human cadaver model of CPR | By using human cadaver, the study  compared tidal volumes between 3 transport ventilators. | All transport ventilators can provide good ventilation even though chest compression considerably decreases tidal volumes. |
| 10 | A Hernández-Tejedor, 2023  [8] | Ventilatory improvement with mechanical ventilator versus bag in non-traumatic out-of-hospital cardiac arrest: SYMEVECA study, phase 1 | A pragmatic prospective nonrandomized trial comparing ABG, patient outcome on OHCA patients between  MV( peak pressure 50 cm-H_2_O) and BVM. | MV had better ventilation than BVM,  but no difference between outcomes. |
| 11 | T Tangpaisarn, 2023  [18] | The effects of mechanical versus bag-valve ventilation on gas exchange during cardiopulmonary resuscitation in emergency department patients: A randomized controlled trial (CPR-VENT) | In cardiac arrest patients in ED, the RCT compared ABG parameter, patient outcome and pneumothorax between  MV( peak pressure 60 cm-H_2_O) and BVM. | The ABG parameter, patient outcome and pneumothorax showed no differences between 2 groups. |
| 12 | J Shin, 2024  [19] | Automatic Mechanical Ventilation vs Manual Bag Ventilation During CPR: A Pilot Randomized Controlled Trial | A pilot RCT comparing ABG parameter, ventilation and patient outcome in OHCA patients between  MV( peak pressure 70 cm-H_2_O) and BVM. | MV had lower tidal volume (267 vs. 507mL, p<0.001) and lower minute ventilation (3.1 vs. 6.7L p=0.009),  but no difference between outcomes.  No ABG parameters difference between groups. |

# Trial Design

This trial is a pragmatic, open-label, multi-center cluster randomized controlled trial.

## Hypothesis

The hypothesis of the trial was formulated using the PICO framework (Population-Intervention-Comparison-Outcome). The study focused on non-traumatic out-of-hospital cardiac arrest (OHCA) patients in Hsinchu County (Population). It hypothesized that patients treated with advanced airway management and automated pneumatic ventilation (Intervention) would achieve a higher rate of return of spontaneous circulation (ROSC) (Outcome) compared to those who received bag-valve-mask ventilation (Comparison).

## Objective

The primary objective of this trial is to evaluate the clinical effectiveness of the automated pneumatic ventilation (APV) method in prehospital resuscitation of out-of-hospital cardiac arrest (OHCA) compared to the bag-valve-mask (BVM) method. The primary outcome is any return of spontaneous circulation (ROSC).

The secondary objectives include:

1. Comparing the ventilation parameters between the APV and BVM methods.
2. Evaluating the overall resuscitation quality in the APV and BVM groups.
3. Comparing the satisfaction levels of emergency medical technicians between the APV and BVM groups.

## Inclusion criteria

Adult out-of-hospital cardiac arrest with attempted resuscitation by the Hsin-chu County Emergency Medical Service.

## Exclusion criteria

- - 1. Pregnancy
    2. Died from trauma
    3. ROSC before EMS arrival
    4. Dead on arrival (reaching conditions such as decomposition, rigor mortis severe burns, decapitation, evisceration, or trunk fracture)
    5. Refusal of medical treatment by family members
    6. No placement of an advanced airway throughout the procedure

## Trial setting

The EMS teams in Hsinchu County will be enrolled in the study once the necessary equipment, including the APV and Zoll X-series, is ready.

The EMT-P personnel are trained and authorized to perform endotracheal intubation (ETT) and supraglottic airway (SGA) insertion, while EMT-2 personnel are trained and authorized to perform SGA insertion. According to the regulations of the Taiwan Ministry of Health and Welfare, EMT-2 personnel must complete a minimum of 280 hours of training, while EMT-P personnel are required to complete 1,280 hours of training.

Hsinchu County is a rapidly growing area with a population of approximately 560,000 people. The county covers an area of 1,427 km² and is known for its significant contributions to Taiwan's technology sector. The population is predominantly Taiwanese, with a mix of Hakka, Han Chinese, and other ethnic groups.

The CONSORT flow diagram of this trial is illustrated in Figure 1.

Figure 1. CONSORT flow diagram of the trial


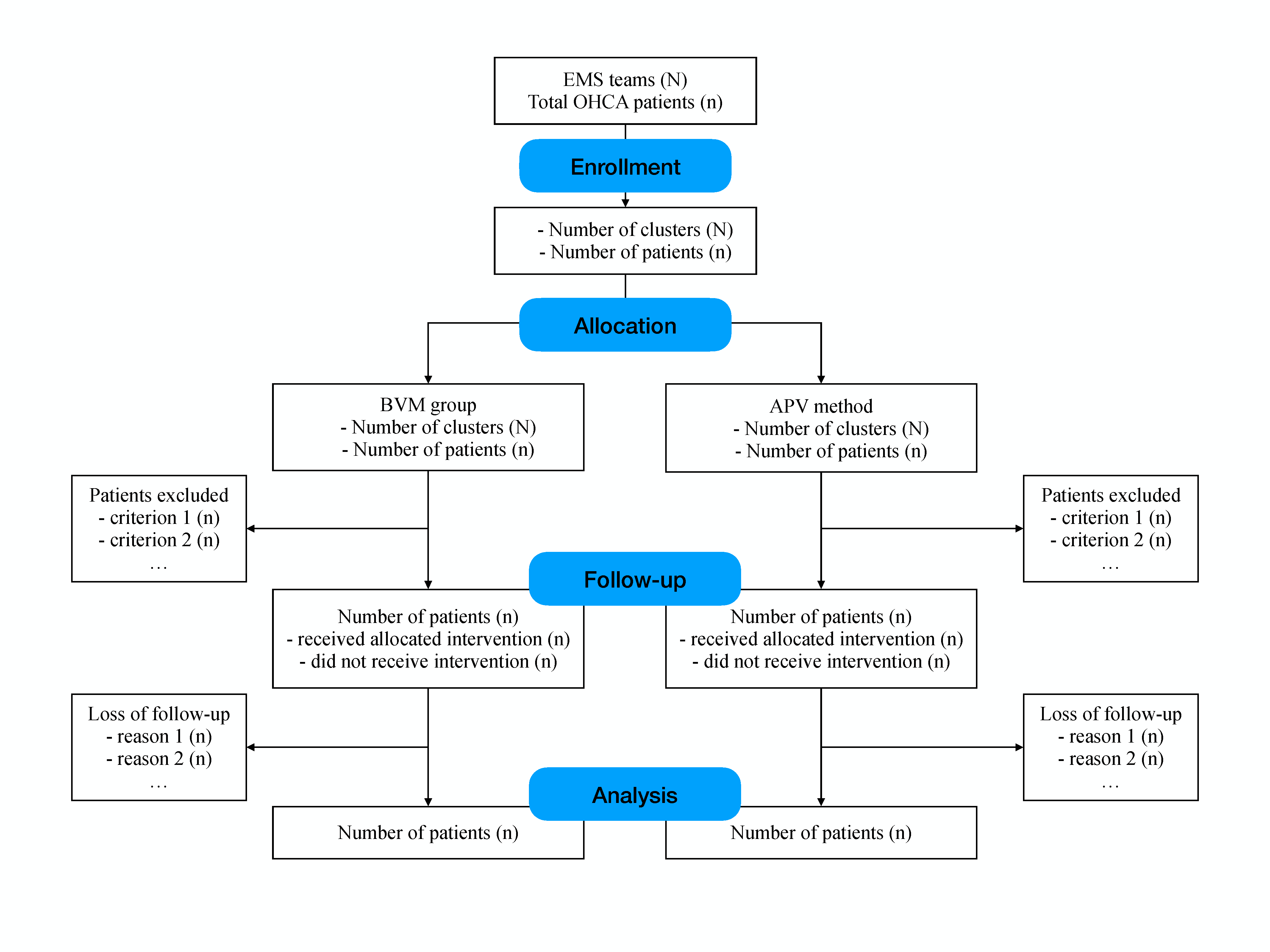


## Randomization and blinding

The participating EMS teams will be divided into two randomized clusters. Each cluster will be assigned to either the APV or BVM group. The duration of a cluster is 14 days (two weeks). The study center will instruct the clusters to alternate between APV and BVM according to a sequence determined by random allocation.

The randomization process was done by a research assistant. The sequence was generated using tool by Sealed Envelope Ltd. 2022 [20]. The formula this program used is based on the textbook *Clinical Trials: A Practical Approach* [21]. In the program, the seed is generated by a random table by Microsoft Excel. Each block contains 6 clusters. However, the EMTs are not aware of the randomization and allocation method.

The allocation sequence will be revealed until three days before the next cluster begins via social network application.

Blinding of the EMTs or hospital staff is not feasible because the ventilation equipment used in the study cannot be blinded

## Pre-trial training program

Before the trial begins, all EMS teams will participate in a 2-hour training program that covers the study protocol and procedures. Following this training, the EMS teams will undergo a 2-month pilot phase to ensure all members are fully familiar with the trial protocol. During the pilot phase, the EMS teams will execute the trial protocol; however, the data collected during this period will be used solely for training purposes and procedural adjustments. Any new teams joining the trial must complete the same training and pilot phase before participating.

## Exposure management

- - 1. Intervention group

The intervention group will receive APV for eligible patients, as determined by the selection criteria. The APV will be connected to the advanced airway (either SGA or ETT) in a standardized manner. The APV is set to deliver one breath every six seconds, with a tidal volume of 500-600 mL and a pressure limit of 60 mmHg. All other advanced cardiovascular life support (ACLS) procedures will follow the OHCA operation guidelines. If the APV malfunctions, the EMS team will switch to bag-valve-mask (BVM) ventilation.

- - 1. Control group

The control group will receive BVM ventilation for eligible patients, as determined by the selection criteria. The BVM will be connected to the advanced airway (either SGA or ETT) in the same manner as in the intervention group. The EMT will manually deliver a breath every six seconds.

- - 1. Advanced airway management

The decision to insert an SGA or perform ETT intubation depends on the level of the EMT attending the OHCA rescue. Only EMT-P personnel are authorized to perform ETT intubation. If an SGA is initially inserted, switching to ETT during the same dispatch is not permitted. If two attempts at ETT insertion fail, an SGA should be used instead.

## Outcome measurement

- - 1. Primary outcome

The primary outcome of the trial is any ROSC (defined as prehospital ROSC or ROSC achieved after in-hospital resuscitation).

- - 1. Secondary outcomes

The secondary outcomes include the following:

- Prehospital ROSC: ROSC before hospital arrival
- 24-hour survival
- Survival to discharge
- Neurological function on discharge
- Ventilation rate in prehospital resuscitation
- Tidal volume in prehospital resuscitation
- End-tidal CO2 level in prehospital resuscitation
- Chest compression fraction in prehospital resuscitation
- Intravenous catheter insertion in prehospital resuscitation
- Epinephrine injection in prehospital resuscitation
- Satisfaction of EMT in prehospital resuscitation (Appendix 2)

## Study procedures and workflow

- - 1. Weekly Group Assignment Announcement

Every Friday evening, the group allocation results are announced via the study group's communication app. If assigned to the intervention group, the paramedics must ensure that the APV device is functioning properly and place it in the ambulance. If allocated to the control group, the device should be removed from the ambulance. The new round of group allocation will begin on Monday at 8 AM.

- - 1. OHCA rescue

When the dispatch center reports an OHCA case, an OHCA is identified on scene, or a patient experiences cardiac arrest during transportation to the hospital, the EMTs will confirm whether the case meets the inclusion criteria. The EMTs will then perform ACLS and determine which advanced airway (ETT or SGA) to use based on the situation at the scene.

The standard ACLS procedure in Hsin-chu county is provided in Appendix 1.

- - 1. Device Setup After Establishing Advanced Airway

After establishing the advanced airway (SGA or ETT), the following equipment should be connected under the following sequence:

Patient → Endotracheal tube or supraglottic airway → HEPA filter → AccuVent sensor → End-tidal CO2 sensor → Automatic ventilator → Oxygen tank (Figure 1).

- - 1. Data Entry After Rescue

After completing the rescue, upon returning to the fire department, the EMT should log the case data into an Excel form and upload the rescue reporting form. Additionally, they should complete the satisfaction questionnaire after every successful patient recruit.

These data are stored in a secure database.

- - 1. Data Export from Zoll X-series

A designated staff member will export the case’s ventilation and chest compression physiological data from the Zoll X-series monitor and then upload to a secured database.

Figure 2. Equipment connection for enrolled OHCA patients.


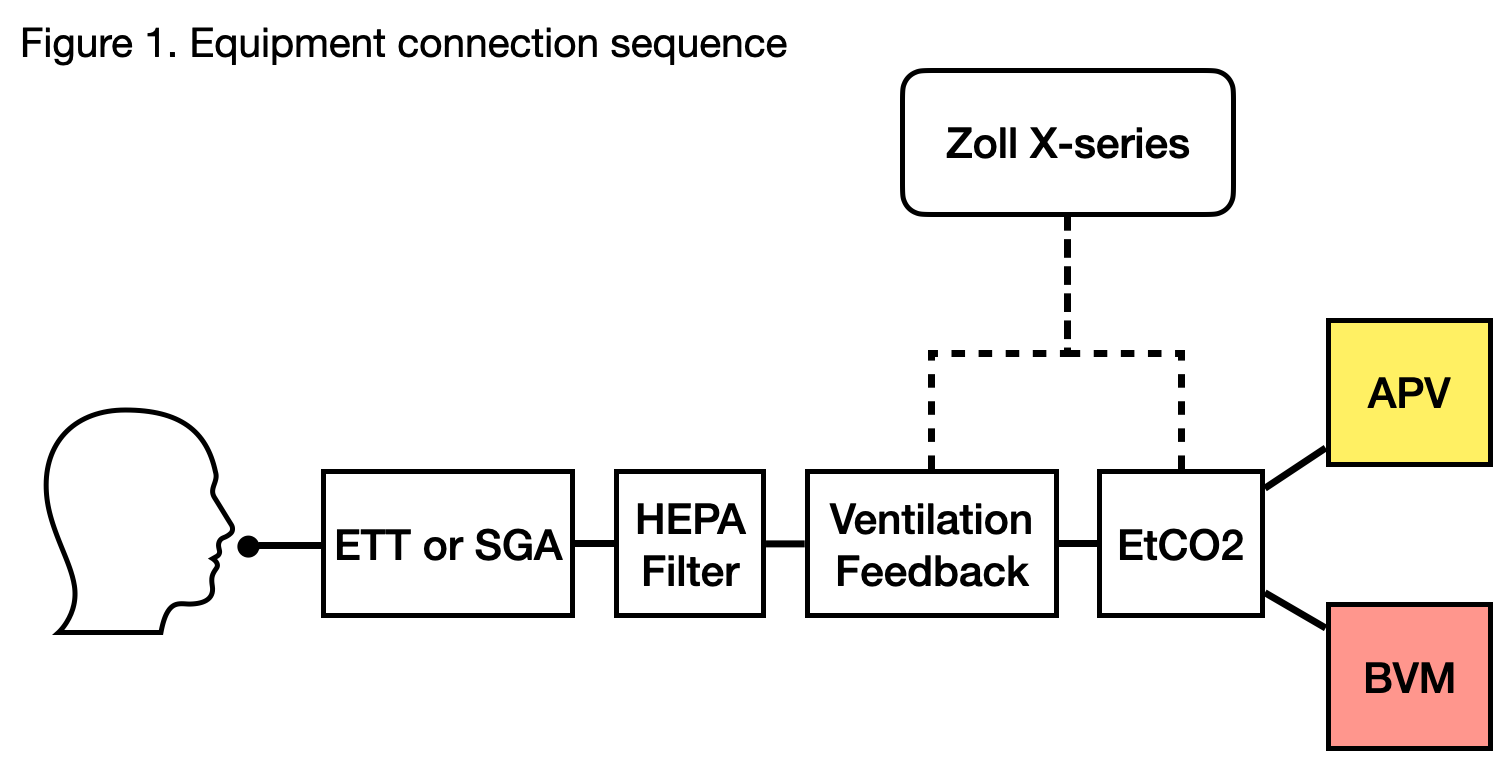


# Sample Size Estimation

In the original version of sample size calculation, we chose a secondary outcome (ventilation rate) to be the reference to calculate the sample size. Because we couldn’t find any studies with a similar trial design and primary outcome as our study. The original sample size was 260 patients. However, in August 2024, we identified some comparable studies to guide our calculations more precisely.

We chose the pilot study by Shin et al., which compared automatic mechanical ventilation (MV) with manual bag ventilation (BV) during CPR [19]. In that study, the primary outcome, any ROSC, was achieved in 56.7% of the MV group and 43.3% of the BV group—values closely aligning with our expected ROSC rates. Thus, we conducted an initial power analysis in G*Power 3.1 using the following parameters: Z test family, difference between two independent proportions, two-tailed test, proportions of 0.567 and 0.433, alpha of 0.05, power of 0.8, and equal allocation (1:1). This analysis suggested 218 patients per arm, totaling 436.

To account for the clustering effect due to randomization at the EMS team level, we included an intraclass correlation coefficient (ICC). Our EMS teams are expected to recruit 3 patients per cluster (over 14 days). Given the substantial variability in OHCA patient conditions, we assumed a low ICC of 0.06. The design effect was therefore calculated as 1+(3−1) ×0.06=1.12, adjusting our sample size to 488. Finally, with an estimated 5% dropout rate, the final required sample size was increased to 514 patients.

Figure 3. Sample size calculation by G*Power 3.1


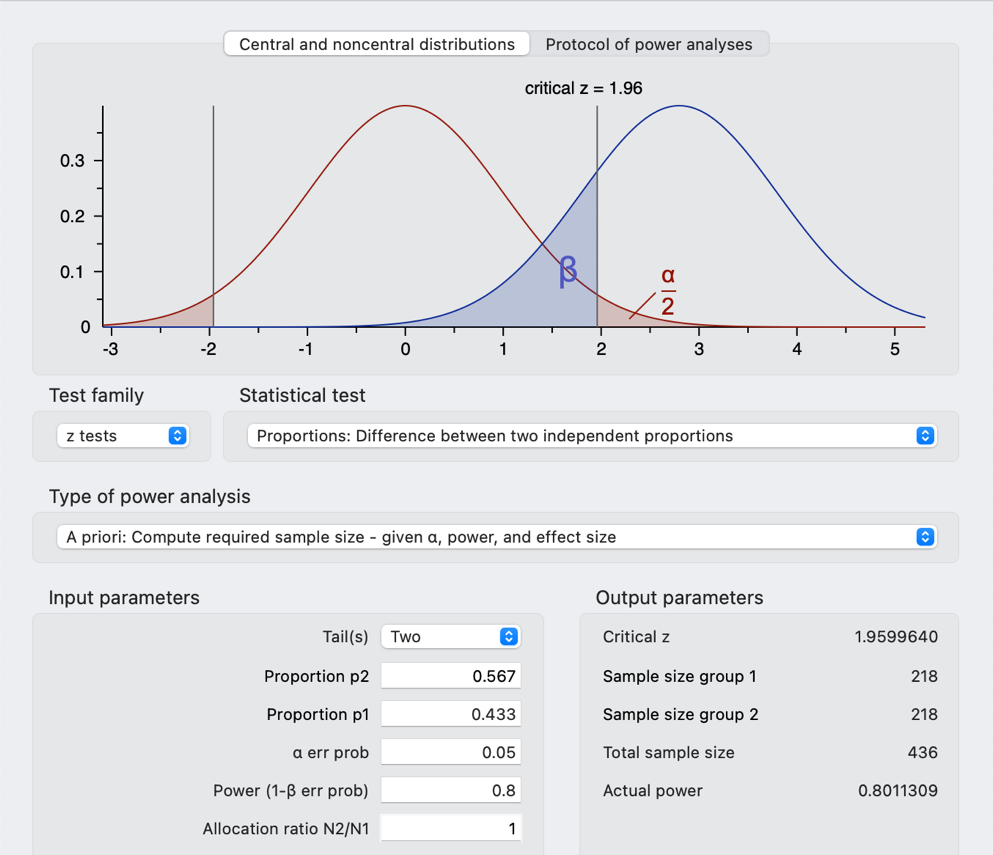


# Data and Safety Monitoring Boards and Interim Analysis

This study has established a Data and Safety Monitoring Board (DSMB) in accordance with the standards of the National Institutes of Health (NIH). The DSMB will review the completeness of the data and monitor for any obvious abnormalities that could compromise the patients' safety every six months. Additionally, when enrollment reaches half of the target sample size (130 participants), the board will independently conduct an interim analysis. If any concerning situations arise, the board will consult with the principal investigator to determine whether the study should be suspended. The DSMB meetings will be held every six months.

## Interim analysis and stopping criteria

- - 1. All ROSC (Return of Spontaneous Circulation) Events

If the number of ROSC events in one group significantly exceeds that of the other group, as determined by a chi-square test in a 2x2 table, and if this difference reaches statistical significance (p-value < 0.05), the study may be terminated.

- - 1. Adverse Events

The known potential adverse event related to the ventilator that could be assessed in this study is barotrauma (pressure-related injury to the airway). However, considering that the study subjects are OHCA (out-of-hospital cardiac arrest) patients, and not all of them can be evaluated for the occurrence of barotrauma under standard medical practice, the threshold for adverse events in this study is set at 5%. If more than 15 cases of potential barotrauma (e.g., pneumothorax) occur in the experimental group, the study must be terminated.

- - 1. Other criteria
       1. Changes in government emergency medical services policies
       2. The APV is found to be harmful to humans during the study
       3. Major compromise to the EMS work due to the study

# Data Collection and Follow-up

Data will be collected in the REDCap database set up at National Taiwan University Hospital. The following variables will be gathered from the rescue reporting form: patient demographics (age and sex), pre-existing comorbidities (hypertension, diabetes mellitus, COPD, asthma, liver disease, kidney disease, heart disease, seizure, psychotic disorder, and cancer), arrest characteristics (witnessed arrest and bystander CPR), arrest location (household, workplace, public space, and nursing home), and EMT-performed procedures (oral/nasal airway, SGA, ETT, mechanical CPR, AED, defibrillation, IO/IV catheter, epinephrine injection, and normal saline infusion). Prehospital ROSC will also be reported on the form. These data will be collected on a weekly basis.

Other prognostic outcomes (any ROSC, 24-hour survival, survival to discharge, and neurological function at discharge) will be collected from the reports of the hospitals to which the patients are sent. Follow-up frequency for these outcomes will be one month.

Ventilation and chest compression feedback data will be exported from the Zoll X-series in .csv format. These files will be uploaded to the REDCap database on a weekly basis.

# Data Management

Data collected during the study will be entered into a secure, password-protected electronic database. The following procedures will be implemented to ensure data quality, confidentiality, and integrity:

1. **Data Entry and Coding**: Data will be double-entered by two independent data entry personnel to minimize data entry errors. Each data entry will then be cross-checked for discrepancies, which will be resolved by the direction of the trial. All data will be anonymized and coded prior to entry, with identifiers kept in a separate, secure file to maintain participant confidentiality.
2. **Data Quality Assurance**: Range checks will be implemented for numerical data fields to identify out-of-range or erroneous values. Additionally, logical checks (e.g., comparing date fields for chronological consistency) will be performed. Any identified data inconsistencies will be flagged for review, and corrections will be documented in an audit trail of the REDCap database.
3. **Data Security**: The database will be hosted on a secure server with restricted access provided by REDCap, limited to authorized study personnel.
4. **Data Storage**: All data will be stored in compliance with relevant regulations and institutional guidelines of National Taiwan University Hospital and Hsin-Chu County Fire Department. Upon study completion, data will be archived securely for a period of five years, in line with institutional and regulatory requirements. After this retention period, data will be anonymized and securely disposed of as per institutional policies.

# Analytic Plan

Statistical Package for the Social Sciences (version 26.0; IBM Corp., Armonk, NY, USA) will be used for the statistical analyses in this study. Intention to treat analysis will be adopted for comparing outcomes between interventions. Firstly, descriptive statistics of demographics (Table 1), ventilation parameters (Table 2), and outcomes (Table 3) among APV group and BVM group will be presented as number (percentage) for categorical variables and median (interquartile range) for continuous variables. Categorical variables will be compared using Pearson’s chi-square test or Fisher’s exact test, while continuous variables will be compared using Mann–Whitney U test. Secondly, multivariable logistic regression will be conducted to investigate the association of different ventilation interventions and patients’ outcomes, adjusting for potential confounders. Subgroup analysis, presented as a forest plot, will also be performed to investigate the impact of different interventions on outcomes in different subgroups. We will also compare the adjusted absolute difference between ventilation parameters measured in the APV and BVM group using the linear mixed-effects model (LMM). For the LMM, variables including demographics, prehospital information, resuscitation managements were included as fixed effects, whereas each patient will be modeled as a random effect. Two tail p-values <0.05 will be considered as statistically significant and will be corrected with Bonferroni correction in post-hoc analyses.

# Ethics

1. **Research ethics approval**: The study was approved by the Institutional Review Board of National Taiwan University Hospital (no. 202304132RINB).
2. **Protocol amendments**: Any modification of the protocol of the study will be discussed with the Institutional Review Board and execute only after being approved.
3. **Informed consent**: The PIVOT trial recruits unconscious patients who have experienced OHCA, and the prehospital treatment is time-sensitive. Therefore, it is challenging and impractical for EMS technicians to obtain informed consent from either the patient or their family members before initiating ACLS and trial interventions. Following discussions with the Institutional Review Board (IRB), the trial has been approved for a waiver of informed consent for all participants.

# Dummy Tables

| Table 1. Baseline characteristics | | |
| --- | --- | --- |
|  | APV | BVM |
|  | (n=XXX) | (n=XXX) |
| Age, years (mean ± SD) | XX ± XX | XX ± XX |
| Males, n (%) | XX (XX.X%) | XX (XX.X%) |
| Pre-existing comorbidity |  |  |
| Diabetic mellitus, n (%) | XX (XX.X%) | XX (XX.X%) |
| Hypertension, n (%) | XX (XX.X%) | XX (XX.X%) |
| Cancer, n (%) | XX (XX.X%) | XX (XX.X%) |
| COPD, n (%) | XX (XX.X%) | XX (XX.X%) |
| Asthma, n (%) | XX (XX.X%) | XX (XX.X%) |
| Stroke, n (%) | XX (XX.X%) | XX (XX.X%) |
| Liver disease, n (%) | XX (XX.X%) | XX (XX.X%) |
| Kidney disease, n (%) | XX (XX.X%) | XX (XX.X%) |
| Heart disease, n (%) | XX (XX.X%) | XX (XX.X%) |
| Seizure, n (%) | XX (XX.X%) | XX (XX.X%) |
| Psychotic disorder, n (%) | XX (XX.X%) | XX (XX.X%) |
| Witnessed arrest, n (%) | XX (XX.X%) | XX (XX.X%) |
| Bystander CPR, n (%) | XX (XX.X%) | XX (XX.X%) |
| Arrest location |  |  |
| Household, n (%) | XX (XX.X%) | XX (XX.X%) |
| Working place, n (%) | XX (XX.X%) | XX (XX.X%) |
| Public space, n (%) | XX (XX.X%) | XX (XX.X%) |
| Nursing home, n (%) | XX (XX.X%) | XX (XX.X%) |
| Others, n (%) | XX (XX.X%) | XX (XX.X%) |
| Oral airway, n (%) | XX (XX.X%) | XX (XX.X%) |
| Nasal airway, n (%) | XX (XX.X%) | XX (XX.X%) |
| SGA, n (%) | XX (XX.X%) | XX (XX.X%) |
| ETT, n (%) | XX (XX.X%) | XX (XX.X%) |
| Mechanical CPR, n (%) | XX (XX.X%) | XX (XX.X%) |
| AED, n (%) | XX (XX.X%) | XX (XX.X%) |
| Defibrillation, n (%) | XX (XX.X%) | XX (XX.X%) |
| IO, n (%) | XX (XX.X%) | XX (XX.X%) |
| IV, n (%) | XX (XX.X%) | XX (XX.X%) |
| Epinephrine, n (%) | XX (XX.X%) | XX (XX.X%) |
| Normal saline, n (%) | XX (XX.X%) | XX (XX.X%) |

| Table 2. Ventilation parameters between patients in APV and BVM group | | |
| --- | --- | --- |
|  | APV | BVM |
|  | (n=XXX) | (n=XXX) |
| Ventilation rate, bpm (median ± IQR) | XX ± XX | XX ± XX |
| Tidal volume, mL (median ± IQR) | XX ± XX | XX ± XX |
| EtCO2, mmHg (median ± IQR) | XX ± XX | XX ± XX |

| Table 3. Outcome comparison between patients in APV and BVM group | | |
| --- | --- | --- |
|  | APV | BVM |
|  | (n=XXX) | (n=XXX) |
| Prehospital ROSC, n (%) | XX (XX.X%) | XX (XX.X%) |
| Any ROSC, n (%) | XX (XX.X%) | XX (XX.X%) |
| 24-hour survival, n (%) | XX (XX.X%) | XX (XX.X%) |
| Survival to hospital discharge, n (%) | XX (XX.X%) | XX (XX.X%) |
| Good neurological function after discharge, n (%) | XX (XX.X%) | XX (XX.X%) |

# Timeline

## First year working plan (2023)

| No | Process | Time (month) | | | | | | | | | | | |
| --- | --- | --- | --- | --- | --- | --- | --- | --- | --- | --- | --- | --- | --- |
|  |  | 01 | 02 | 03 | 04 | 05 | 06 | 07 | 08 | 09 | 10 | 11 | 12 |
| 1 | Proposal |  |  |  |  |  |  |  |  |  |  |  |  |
| 2 | IRB process |  |  |  |  |  |  |  |  |  |  |  |  |
| 3 | Data collect |  |  |  |  |  |  |  |  |  |  |  |  |

## Second year working plan (2024)

| No | Process | Time (month) | | | | | | | | | | | |
| --- | --- | --- | --- | --- | --- | --- | --- | --- | --- | --- | --- | --- | --- |
|  |  | 01 | 02 | 03 | 04 | 05 | 06 | 07 | 08 | 09 | 10 | 11 | 12 |
| 1 | Data collect |  |  |  |  |  |  |  |  |  |  |  |  |
| 2 | Interim analysis |  |  |  |  |  |  |  |  |  |  |  |  |

## Third year working plan (2025)

| No | Process | Time (month) | | | | | | | | | | | |
| --- | --- | --- | --- | --- | --- | --- | --- | --- | --- | --- | --- | --- | --- |
|  |  | 01 | 02 | 03 | 04 | 05 | 06 | 07 | 08 | 09 | 10 | 11 | 12 |
| 1 | Data collect |  |  |  |  |  |  |  |  |  |  |  |  |
| 2 | Interim analysis |  |  |  |  |  |  |  |  |  |  |  |  |

## Fourth year working plan (2026)

| No | Process | Time (month) | | | | | | | | | | | |
| --- | --- | --- | --- | --- | --- | --- | --- | --- | --- | --- | --- | --- | --- |
|  |  | 01 | 02 | 03 | 04 | 05 | 06 | 07 | 08 | 09 | 10 | 11 | 12 |
| 1 | Data analysis |  |  |  |  |  |  |  |  |  |  |  |  |
| 2 | Paper writing |  |  |  |  |  |  |  |  |  |  |  |  |
| 3 | Paper submit |  |  |  |  |  |  |  |  |  |  |  |  |

# Reference

1. Yan S, Gan Y, Jiang N, Wang R, Chen Y, Luo Z, et al. The global survival rate among adult out-of-hospital cardiac arrest patients who received cardiopulmonary resuscitation: a systematic review and meta-analysis. Crit Care. 2020;24(1):61. Epub 20200222. doi: 10.1186/s13054-020-2773-2. PubMed PMID: 32087741; PubMed Central PMCID: PMCPMC7036236.

2. Kashef MA, Lotfi AS. Evidence-Based Approach to Out-of-Hospital Cardiac Arrest. Curr Treat Options Cardiovasc Med. 2021;23(6):43. Epub 20210510. doi: 10.1007/s11936-021-00924-3. PubMed PMID: 33994773; PubMed Central PMCID: PMCPMC8107417.

3. Delnoij TSR, Suverein MM, Essers BAB, Hermanides RC, Otterspoor L, Elzo Kraemer CV, et al. Cost-effectiveness of extracorporeal cardiopulmonary resuscitation vs. conventional cardiopulmonary resuscitation in out-of-hospital cardiac arrest: a pre-planned, trial-based economic evaluation. Eur Heart J Acute Cardiovasc Care. 2024;13(6):484-92. doi: 10.1093/ehjacc/zuae050. PubMed PMID: 38652269.

4. Geri G, Gilgan J, Ziegler C, Isaranuwatchai W, Morrison LJ. Costs related to cardiac arrest management: a systematic review protocol. Syst Rev. 2017;6(1):205. Epub 20171017. doi: 10.1186/s13643-017-0599-z. PubMed PMID: 29041982; PubMed Central PMCID: PMCPMC5646160.

5. Rivard MK, Cash RE, Chrzan K, Panchal AR. The Impact of Working Overtime or Multiple Jobs in Emergency Medical Services. Prehosp Emerg Care. 2020;24(5):657-64. Epub 20191220. doi: 10.1080/10903127.2019.1695301. PubMed PMID: 31750761.

6. Ni SA, Carpenter RS, Walker JR, 3rd, Holley J, Brady MF. Emergency Medical Services Responder Manual Ventilation Performance. Prehosp Emerg Care. 2023;27(4):496-500. Epub 20220609. doi: 10.1080/10903127.2022.2066234. PubMed PMID: 35442149.

7. Panchal AR, Bartos JA, Cabañas JG, Donnino MW, Drennan IR, Hirsch KG, et al. Part 3: Adult Basic and Advanced Life Support: 2020 American Heart Association Guidelines for Cardiopulmonary Resuscitation and Emergency Cardiovascular Care. Circulation. 2020;142(16_suppl_2):S366-s468. Epub 20201021. doi: 10.1161/cir.0000000000000916. PubMed PMID: 33081529.

8. Hernández-Tejedor A, González Puebla V, Corral Torres E, Benito Sánchez A, Pinilla López R, Galán Calategui MD. Ventilatory improvement with mechanical ventilator versus bag in non-traumatic out-of-hospital cardiac arrest: SYMEVECA study, phase 1. Resuscitation. 2023;192:109965. Epub 20230912. doi: 10.1016/j.resuscitation.2023.109965. PubMed PMID: 37709164.

9. Weiss SJ, Ernst AA, Jones R, Ong M, Filbrun T, Augustin C, et al. Automatic transport ventilator versus bag valve in the EMS setting: a prospective, randomized trial. South Med J. 2005;98(10):970-6. doi: 10.1097/01.smj.0000182177.01436.70. PubMed PMID: 16295811.

10. Johannigman JA, Branson RD, Johnson DJ, Davis K, Jr., Hurst JM. Out-of-hospital ventilation: bag--valve device vs transport ventilator. Acad Emerg Med. 1995;2(8):719-24. doi: 10.1111/j.1553-2712.1995.tb03624.x. PubMed PMID: 7584751.

11. Osterwalder JJ, Schuhwerk W. Effectiveness of mask ventilation in a training mannikin. A comparison between the Oxylator EM100 and the bag-valve device. Resuscitation. 1998;36(1):23-7. doi: 10.1016/s0300-9572(97)00091-9. PubMed PMID: 9547840.

12. Barnes TA, Catino ME, Burns EC, Chan WK, Ghazarian G, Henneberg WR, et al. Comparison of an oxygen-powered flow-limited resuscitator to manual ventilation with an adult 1,000-mL self-inflating bag. Respir Care. 2005;50(11):1445-50. PubMed PMID: 16253151.

13. Salas N, Wisor B, Agazio J, Branson R, Austin PN. Comparison of ventilation and cardiac compressions using the Impact Model 730 automatic transport ventilator compared to a conventional bag valve with a facemask in a model of adult cardiopulmonary arrest. Resuscitation. 2007;74(1):94-101. Epub 20070206. doi: 10.1016/j.resuscitation.2006.01.023. PubMed PMID: 17287062.

14. Hu X, Ramadeen A, Laurent G, So PP, Baig E, Hare GM, et al. The effects of an automatic, low pressure and constant flow ventilation device versus manual ventilation during cardiovascular resuscitation in a porcine model of cardiac arrest. Resuscitation. 2013;84(8):1150-5. Epub 20130227. doi: 10.1016/j.resuscitation.2013.02.017. PubMed PMID: 23454260.

15. Kill C, Hahn O, Dietz F, Neuhaus C, Schwarz S, Mahling R, et al. Mechanical ventilation during cardiopulmonary resuscitation with intermittent positive-pressure ventilation, bilevel ventilation, or chest compression synchronized ventilation in a pig model. Crit Care Med. 2014;42(2):e89-95. doi: 10.1097/CCM.0b013e3182a63fa0. PubMed PMID: 24158168.

16. Neumamm LBA, Jardim-Neto AC, Motta-Ribeiro GC. Empirical evidence for safety of mechanical ventilation during simulated cardiopulmonary resuscitation on a physical model. Am J Emerg Med. 2021;48:312-5. Epub 20210701. doi: 10.1016/j.ajem.2021.06.062. PubMed PMID: 34265507.

17. Orlob S, Wittig J, Hobisch C, Auinger D, Honnef G, Fellinger T, et al. Reliability of mechanical ventilation during continuous chest compressions: a crossover study of transport ventilators in a human cadaver model of CPR. Scand J Trauma Resusc Emerg Med. 2021;29(1):102. Epub 20210728. doi: 10.1186/s13049-021-00921-2. PubMed PMID: 34321068; PubMed Central PMCID: PMCPMC8316711.

18. Tangpaisarn T, Tosibphanom J, Sata R, Kotruchin P, Drumheller B, Phungoen P. The effects of mechanical versus bag-valve ventilation on gas exchange during cardiopulmonary resuscitation in emergency department patients: A randomized controlled trial (CPR-VENT). Resuscitation. 2023;193:109966. Epub 20230912. doi: 10.1016/j.resuscitation.2023.109966. PubMed PMID: 37709163.

19. Shin J, Lee HJ, Jin KN, Shin JH, You KM, Lee SGW, et al. Automatic Mechanical Ventilation vs Manual Bag Ventilation During CPR: A Pilot Randomized Controlled Trial. Chest. 2024;166(2):311-20. Epub 20240218. doi: 10.1016/j.chest.2024.02.020. PubMed PMID: 38373673.

20. Ltd. SE. Create a blocked randomisation list. 2022.

21. Pocock SJ. Methods of Randomization. Clinical Trials2013. p. 66-89.
